# Mitochondrial Genomic Evidence of Selective Constraints in Small-Bodied Terrestrial Cetartiodactyla

**DOI:** 10.3390/ani14101434

**Published:** 2024-05-10

**Authors:** Xuesong Mei, Xibao Wang, Xiaoyang Wu, Guangshuai Liu, Yao Chen, Shengyang Zhou, Yongquan Shang, Zhao Liu, Xiufeng Yang, Weilai Sha, Honghai Zhang

**Affiliations:** School of Life Science, Qufu Normal University, Qufu 273165, China; meixsunique@126.com (X.M.);

**Keywords:** Cetartiodactyla, mitochondrial genomes, body size, selective constraints

## Abstract

**Simple Summary:**

Body size is undoubtedly one of the most critical characteristics that serves as a reliable predictor of an organism’s physiological rates and life-history traits. Notably, we observe a decrease in metabolic rate per unit mass with increasing body size. Given that molecular alterations in mitochondrial genes respond to changes in energy demands, significant variations in metabolic rates across different body sizes may exert an influence on mitochondrial evolution. The terrestrial Cetartiodactyla provides an exceptional model for investigating this issue, owing to its remarkable range of body sizes that spans from less than 2 kg to over 1200 kg. Our findings suggest that mitochondrial DNA protein-coding genes in small-bodied species have undergone a more intense purifying selection. In summary, our research contributes to a deeper understanding of the molecular evolutionary patterns of mitochondria, highlighting the interplay between genomic evolution and phenotypic traits.

**Abstract:**

Body size may drive the molecular evolution of mitochondrial genes in response to changes in energy requirements across species of different sizes. In this study, we perform selection pressure analysis and phylogenetic independent contrasts (PIC) to investigate the association between molecular evolution of mitochondrial genome protein-coding genes (mtDNA PCGs) and body size in terrestrial Cetartiodactyla. Employing selection pressure analysis, we observe that the average non-synonymous/synonymous substitution rate ratio (ω) of mtDNA PCGs is significantly reduced in small-bodied species relative to their medium and large counterparts. PIC analysis further confirms that ω values are positively correlated with body size (R^2^ = 0.162, *p* = 0.0016). Our results suggest that mtDNA PCGs of small-bodied species experience much stronger purifying selection as they need to maintain a heightened metabolic rate. On the other hand, larger-bodied species may face less stringent selective pressures on their mtDNA PCGs, potentially due to reduced relative energy expenditure per unit mass. Furthermore, we identify several genes that undergo positive selection, possibly linked to species adaptation to specific environments. Therefore, despite purifying selection being the predominant force in the evolution of mtDNA PCGs, positive selection can also occur during the process of adaptive evolution.

## 1. Introduction

The mitochondrion, a crucial organelle present in virtually all eukaryotic cells’ cytoplasm, plays a pivotal role in generating energy for diverse cellular activities [1,2]. Surrounded by two membranes, this tiny powerhouse produces approximately 95% of the cell’s energy in the form of adenosine triphosphate (ATP) and harbors its own genome (mtDNA) as well as a relatively independent protein synthesis system [3,4]. In mammals, mtDNA is a closed circular double-stranded molecule that spans approximately 16.5 kb and encodes 37 genes, including 2 rRNA subunits, 22 tRNAs, and 13 proteins involved in oxidative phosphorylation (OXPHOS) [5,6]. Due to its small size, lack of recombination, maternal inheritance, and high substitution rates, mtDNA is widely applied in evolutionary and conservation studies [7,8].

Variations in mitochondrial genome protein-coding genes (mtDNA PCGs) can directly influence metabolic performance, and therefore it is highly sensitive to heat and energy-related selective pressure [9]. How various selective pressures influence mtDNA has attracted considerable attention from researchers. Due to changes in lifestyle, domesticated animals have easier access to food resources, resulting in a reduced need for high energy acquisition efficiency. Many studies have proven that compared to their closely related species, domesticated animals experienced a relaxation of selective constraints on their mtDNA [10,11,12]. Locomotion is highly energy-consuming, therefore species with strong locomotive ability require a more active metabolism than those with weaker abilities. Based on this perspective, strong locomotive species may experience stronger selective constraints on their mtDNA. This phenomenon was observed in strong locomotive mammalian species, volant birds and flying insects [13,14,15,16]. On the other hand, despite strong functional constraints, mtDNA genes have been shown to display evidence of adaptive evolution in numerous studies. For example, researchers examined the molecular evolution of mtDNA PCGs in seven caviomorph rodents, revealing that the evolution of these genes could be linked to metabolic adaptations to low O_2_ environments [17]. Bats are unique mammals that have developed flight as their primary mode of locomotion. Through resequencing and analysis of 77 OXPHOS genes, researchers discovered that both mitochondrial and nuclear OXPHOS genes exhibited evidence of adaptive evolution along the common ancestral branch of bats [18]. These findings support the notion that mtDNA PCGs are involved in energy metabolism are targets of natural selection [19]. Organisms inhabiting high-altitude environments are subjected to severe environmental conditions, including low atmospheric pressure, hypoxia, and intense UV radiation. Some studies have demonstrated that positive selection in mtDNA PCGs plays a strong role in altitude adaptation [20,21,22,23,24]. Collectively, these studies provide evidence that diverse selective pressures exert influence on extensive changes observed in mtDNA.

Body size is a fundamental and crucial phenotypic trait of animals, with variations in body size among species being one of the most noticeable morphological characteristics. The formation of variation in body size is probably a manifestation of the long-term adaptation of organisms to their environment. For instance, small-bodied animals have higher reproductive efficiency, faster development, shorter generation time, and greater adaptability to unstable environments [25,26]. In contrast, large-bodied animals possess their own ecological advantages, such as increased resource accessibility, reduced predation risk, and extended longevity [27]. Overall, body size is a complex feature that is strongly related to factors such as home ranges, habitat, trophic level, and risk of species extinction [28,29]. Actually, body size serves not only as a predictor of ecological parameters but also as a predictor of physiological parameters. Researchers have demonstrated that body size heavily influences metabolic activity and growth rates [30,31]. Because of the high sensitivity of mtDNA evolution to selective pressures related to energy [10], we speculate that body size may also exert an influence on mtDNA as other energy-related selective pressures do. Cetartiodactyla represents an exceptionally diverse and thriving clade of mammals, comprising 23 families, 131 genera and more than 330 extant species [32]. These species have evolved with extreme differences in body size, from less than 2 kg to over 1200 kg in weight [33]. Therefore, this order serves as an excellent model for inferring the impact of body size on mtDNA evolution.

In this study, we initially constructed a robust phylogenetic tree to elucidate the evolution relationships among terrestrial Cetartiodactyla species, recognizing that Whippomorpha species typically attain enormous size and inhabit aquatic environments vastly different from terrestrial ones. Meanwhile, previous studies have demonstrated significant differences in the evolution of mtDNA PCGs between terrestrial and marine Cetartiodactyla [34]. Thus, Whippomorpha is omitted from our analysis. We then examine the molecular evolutionary patterns of mtDNA PCGs in species with different body sizes and their potential roles in environmental adaptation through selective pressure analysis.

## 2. Materials and Methods

### 2.1. MtDNA Sequences and Phenotype Data Collection

We searched all mtDNA sequences available in GenBank for 263 terrestrial Cetartiodactyla species, excluding Whippomorpha. In total, 62 species were excluded due to unavailability, low quality, and lack of annotation in their mtDNA sequences, or extinction. Ultimately, 201 well-annotated mtDNA sequences were acquired. Phenotype data (body mass) were collected from the PanTHERIA database (when available) and by searching the literature [35,36,37,38,39,40]. Relevant information on all species and mtDNA accession numbers are listed in Supplementary Table S1. To detect variations in selective pressures among different body sizes, species were initially classified into three groups based on body mass: large body size group (body mass > 300 kg), small body size group (body mass < 10 kg), and medium body size group (remaining species). The categories also had some minor adjustments according to phylogenetic relationships and body mass. Specifically, if the body mass of a species has led to substantial increases or decreases relative to its sister taxa and was phylogenetically near to that of a sister species clustered in a clade, it can be categorized as either the large or small body size group based on the grouping of its closest relatives. Finally, 59 species out of 201 were included in the selection pressure analysis. The large body size group comprised 23 species, while the small body size group consisted of 19 species. To mitigate the influence of evolutionary relationships, we chose a medium body size group of 17 species, selecting from the sister taxa of either the large or small body size group, for subsequent analysis (Supplementary Table S2).

### 2.2. Phylogenetic Analysis

We extracted 13 mtDNA PCGs from the mitochondrial sequences. Each of the protein sequences of mtDNA PCGs was independently aligned using MUSCLE v3.8.1551 [41], and subsequently converted into codon alignment. Following treatment with Gblocks v0.91b, the alignment outputs were concatenated to generate super sequences (concatenated mtDNA PCGs) for each species, which were utilized in constructing phylogenetic trees [42]. Concatenated mtDNA PCGs were subjected to PartitionFinder v2.1.1 for the selection of optimal partitioning schemes and evolutionary models using a greedy search algorithm and corrected Akaike’s information criterion (AICc) [43]. The software recommended our data be divided into 29 subsets and models (Supplementary Table S3). Bayesian inference (BI) methods with MrBayes v3.2.7 were used to infer the phylogenetic relationships of 201 terrestrial Cetartiodactyla species [44]. The *Canis lupus* was used as an outgroup. Four Markov chain Monte Carlo (MCMC) chains were run simultaneously for 2,000,000 generations with a sampling interval of every 1000 generations.

### 2.3. Selection Pressure Analyses

The selective pressure on concatenated mtDNA PCGs and each mtDNA PCG was inferred by comparing the ratio of non-synonymous to synonymous substitutions (ω = dN/dS) using CODEML in PAML v4.9 [45]. We first utilized the free-ratio model (model = 1, NSsites = 0) to estimate independent ω values of each mtDNA PCG and concatenated mtDNA PCGs for all 59 species. If an unexpectedly small or large ω value (ω < 0.0001 or ω > 10) was observed, this data was excluded from the next analyses and marked as NA data in Supplementary Table S4. Subsequently, a Wilcoxon test was performed to determine if there were significant differences in ω values (each mtDNA PCG) across the three different body size groups (small vs. medium; small vs. large; medium vs. large). We further used the branch model (two-ratio model, model = 2, NSsites = 0; one-ratio model, model = 0, NSsites = 0) to test branch-specific evolutionary rates. Specifically, 3 sets of 1 vs. 2 rate tests were used: (1) Whole tree as 1 rate compared to the small body size group as foreground, rest of tree as background; (2) Whole tree as 1 rate compared to the medium body size group as foreground, rest of tree as background; (3) Whole tree as 1 rate compared to the large body size group as foreground, rest of tree as background. In addition, the branch-site model analyses were conducted using Model A Null (Model = 2, NSsites = 2, fix_omega = 1, omega = 1) and Model A (Model = 2, NSsites = 2, fix_omega = 0, omega = 0) to infer positive selection on the three different body size groups.

### 2.4. Phylogenetic Independent Contrasts Analysis

The potential impact of closely related species on comparative analysis can confound results [20]. To eliminate the influence of evolutionary inertia and investigate the correlation between body size and ω values of mtDNA PCGs, we conducted a phylogenetic independent contrasts (PIC) analysis using the ape package in R [46]. We used Log10-transformed ω values (concatenated mtDNA PCGs) and body mass, while the BI tree was obtained from front phylogenetic analysis in our study. These datasets served as the character data in the process of performing PIC analysis.

## 3. Results

### 3.1. Phylogenetic Analysis

**Figure 1:**
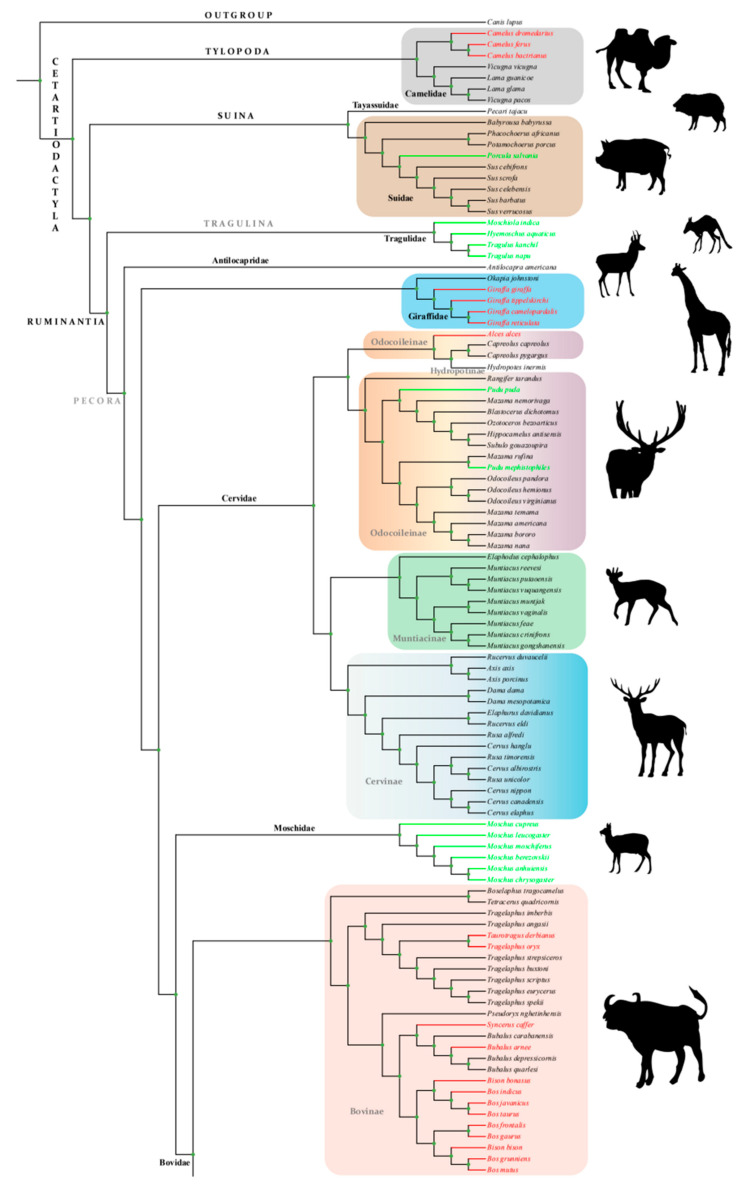

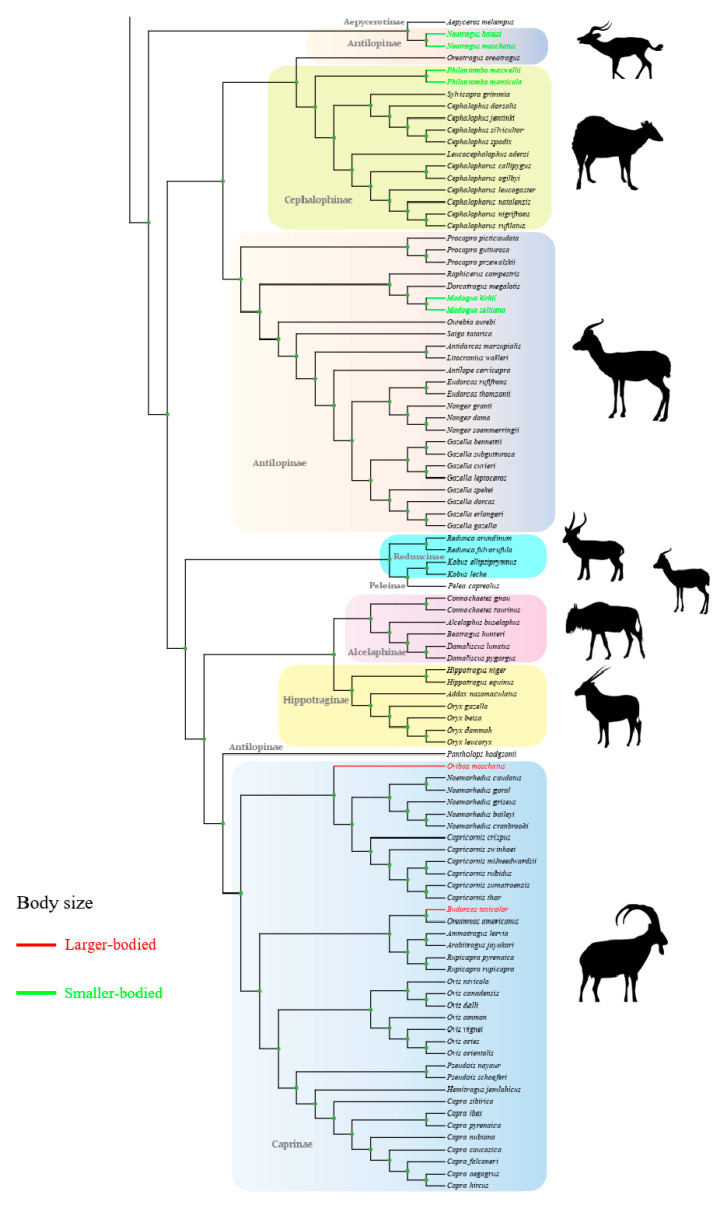
Phylogeny of 201 terrestrial Cetartiodactyla species and the outgroup based on concatenated mtDNA PCGs. The tree uses the Bayesian inference (BI) methods inferred with MrBayes. Larger-bodied and smaller-bodied species are denoted by the colors red and green, respectively.

We utilized concatenated mtDNA PCGs, consisting of 11,175 bp of aligned DNA from 13 mtDNA PCGs, for phylogenetic analysis of 202 species (including 1 outgroup). The BI tree was classified into three major clades: Tylopoda, Suina and Ruminantia (Figure 1), which was consistent with the predecessors’ study [47,48,49]. However, regarding its phylogenetic relationships, the basal position of the order Cetartiodactyla remains controversial. Our research supports Tylopoda as the root position of the phylogenetic tree rather than Suina as reported by some previous studies [32,50].

### 3.2. Selection Pressure Analyses

**Figure 2:**
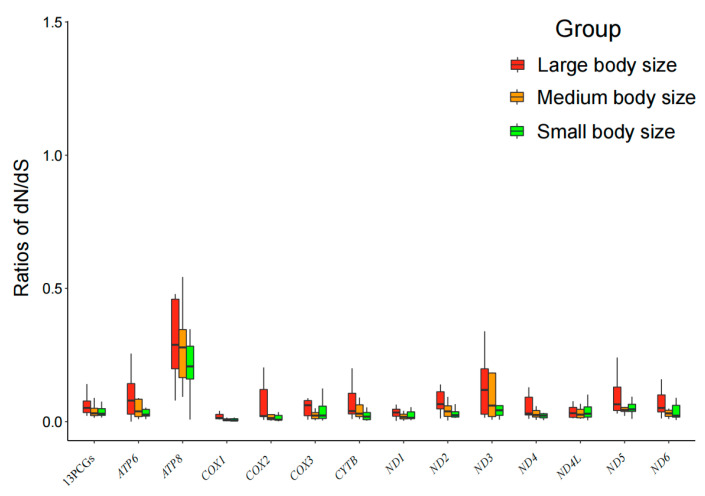
The ω values of concatenated mtDNA PCGs (Marked as “13PCGs” in the figure) and each mtDNA PCG among the three different body size groups based on the free-ratio model.

The ratio of non-synonymous to synonymous substitutions (ω = dN/dS) is widely accepted as an indicator of the strength of selection pressure on protein-coding genes [45]. The ω values of concatenated mtDNA PCGs and each mtDNA PCG were inferred using CodeML in PAML v4.9. In the free-ratio model, most genes exhibited ω values below one across 59 species (Supplementary Table S4), suggesting a conservative evolution of mtDNA PCGs. Nevertheless, we still identified five genes that underwent positive selection: *ATP8* in *Bison bonasus* (ω = 1.43191; large body size group), *Moschus moschiferus* (ω = 1.33399; small body size group) and *Bos indicus* (ω = 1.07309; large body size group); *ND3* in *Lama glama* (ω = 1.07927; medium body size group) and *ND5* in *Bos grunnien* (ω = 1.12243; large body size group). On the other hand, we found that *ATP8* had the highest average ω values, while *COX1* displayed the lowest (Figure 2). These findings imply that, compared with other PCGs in Cetartiodactyla, the substitution rate in *ATP8* is accelerated whereas the rate of evolution of *COX1* might be the slowest.

**Figure 3:**
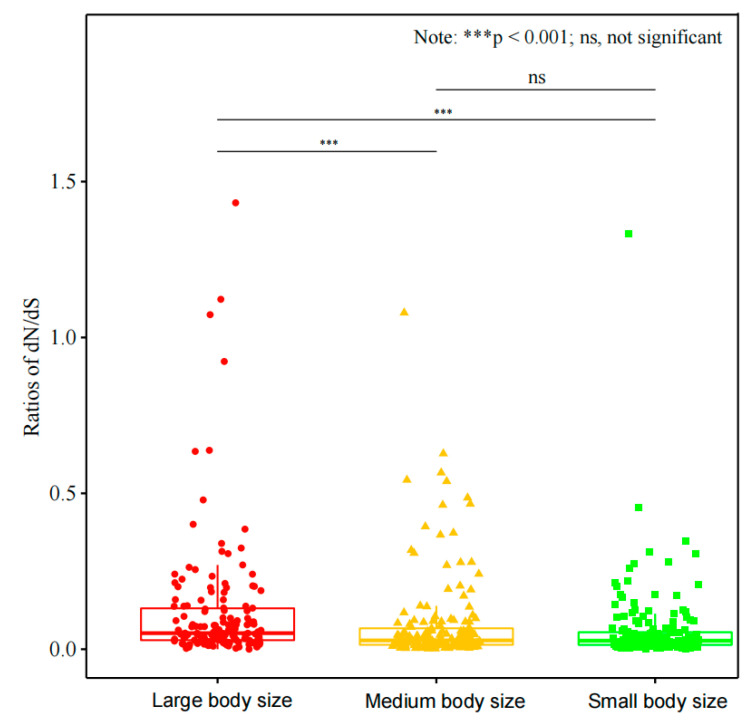
Comparisons of ω values among the three different body size groups based on each mtDNA PCG.

To explore variation in selective pressures across the three different body size groups, we generated a box plot of ω values (each mtDNA PCG) for each group. The results show the mean ω values in the small body size group are significantly lower than those in the large body size group, with no significant difference observed when the former is compared to the medium body size group (Figure 3).

We further used the branch model to investigate whether the three different body size groups were subjected to heterogeneous selective pressures. The ω values of selected branches were estimated using the two-ratio model, while ω values of the background group were calculated under the one-ratio model. The branch model analysis revealed that, seven rapid evolutionary genes were detected in the large body size group (LG): *ATP6* (LG: ω values, 0.05896; BG: ω values, 0.03950), *COX2* (LG: ω values, 0.01725; BG: ω values, 0.00773), *COX3* (LG: ω values, 0.03011; BG: ω values, 0.01707), *CYTB* (LG: ω values, 0.04042; BG: ω values, 0.02452), *ND1* (LG: ω values, 0.02233; BG: ω values, 0.01439), *ND2* (LG: ω values, 0.04675; BG: ω values, 0.03158), and *ND5* (LG: ω values, 0.06504; BG: ω values, 0.04655). Four rapid evolutionary genes were detected in the medium body size group (MG): *COX1* (MG: ω values, 0.00542; BG: ω values, 0.00369), *COX2* (MG: ω values, 0.01175; BG: ω values, 0.00773), *COX3* (MG: ω values, 0.02271; BG: ω values, 0.01707), and *CYTB* (MG: ω values, 0.03466; BG: ω values, 0.02452). However, in the small body size group, no genes exhibiting rapid evolution were identified. Table 1 displays the ω values for each mtDNA PCG among the three different body size groups. In addition, we employed the branch-site model to identify the positive site of each mtDNA PCG, and one positive selection gene was found (Supplementary Table S5).

### 3.3. Phylogenetic Independent Contrasts Analysis

**Figure 4:**
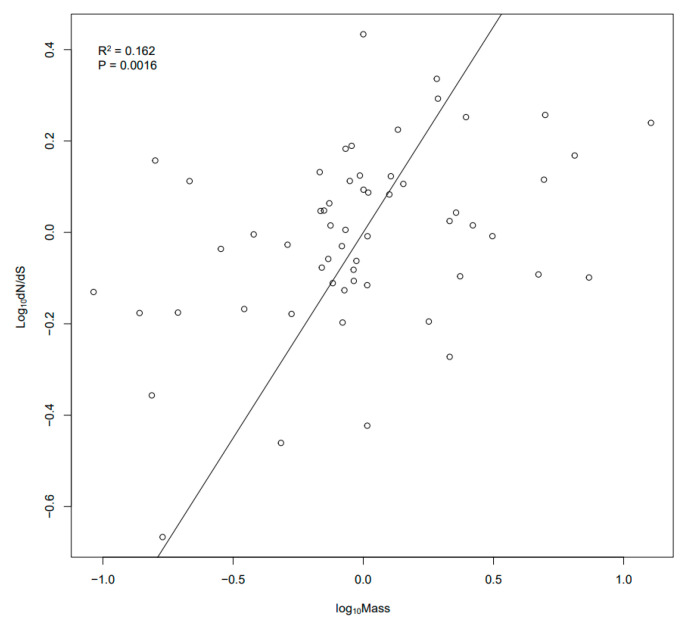
PIC analysis between body mass and ω values of concatenated mtDNA PCGs in 59 studied cetartiodactyla species.

PIC analysis can rid the influence of phylogenetic inertia [20], and a significant positive correlation was observed between ω values of mtDNA PCGs and body mass (R^2^ = 0.162, *p* = 0.0016, Figure 4). This shows that body size has an influence on the evolution of mtDNA PCGs in Cetartiodactyla.

## 4. Discussion

Mitochondria are important cellular organelles that provide up to 95% of energy to almost all eukaryotic cells through oxidative phosphorylation [51,52]. Within mtDNA, all 13 proteins involved in this vital process are under high functional constraints [10]. Metabolic rate increases with the decrease in body size, and the molecular evolution of mitochondrial genes responds to changes in energy requirements [30]. Hence, we hypothesize that the stronger selective constraints may have occurred on mtDNA in small-bodied species due to higher energy consumption. In this study, we focus on the molecular evolution of mtDNA PCGs in relation to body size.

Building a highly credible phylogenetic tree is a necessity and a prerequisite. The topology of the BI tree generated in our study is largely congruent with the evolutionary relationships reported in previous research. While some studies suggested Suina lies basally within the order Cetartiodactyla [32,50], our study recovers Tylopoda at the base of the phylogenetic tree with strong posterior probability ([PP] = 1). Furthermore, phylogenomic analyses based on 110 single-copy nuclear protein-coding genes also supported an early divergence of Tylopoda rather than Suina [48]. These controversies may stem from discrepancies in phylogenetic inference methodologies, molecular markers, and outgroup selection.

Significant differences in metabolic rates across different body sizes may be acting on the evolution of their mitochondria. We select ω values of the terminal branches as an indicator for evaluating selection pressure [45]. In the free-ratio model, most mtDNA PCGs exhibit ω values far less than one (Supplementary Table S4), indicating that these genes are under purifying selection and suggesting conservative evolution of mitochondrial genes. Purifying selection plays a crucial role in shaping mtDNA sequence evolution by exerting strong purification effects on mutations within mtDNA PCGs [53]. However, we still find five genes that undergo positive selection. We infer that these genes may be associated with the adaptation of species to their environment. Interestingly, we observe three species (*Bison bonasus*, *Lama glama*, and *Bos grunnien*) inhabit high-altitude regions. Harsh environments present ecological challenges to them, including elevated levels of solar radiation, low air temperatures, and reduced atmospheric pressure [54]. Thus, our detection of positively selected genes suggests a possible signal of mitochondrial adaptation to a plateau environment.

Different mtDNA PCGs may experience different selections. The results reveal a considerable degree of variation in ω values across 13 mtDNA PCGs (Figure 2). We observe that *ATP8* exhibits the highest average ω values among all mtDNA PCGs studied, indicating a faster evolution rate. *ATP8* is involved in encoding a subunit of ATP synthase, which plays a crucial role in the final stages of cellular respiration [55,56]. The higher ω value in *ATP8* may allow for slightly more beneficial amino acid substitutions, which can help species adapt to diverse ecological niches [57]. Conversely, *COX1* displays the lowest average ω values, suggesting a slower evolutionary pace than its counterparts. One possible explanation is that *COX1* may have more specialized roles compared to other genes, or its mutation could significantly impact the survival and reproduction of the species. Therefore, it is plausible to assume that certain genes have undergone more rigorous selective pressures to eliminate deleterious mutations and maintain their functionality [13]. In brief, even genes that are closely related can exhibit significant variations in their rates and patterns of change over time.

Based on the free-ratio, mean ω values of mtDNA PCGs in the small body size group are significantly lower than large. Preliminary research findings suggest that body size possibly had an impact on the evolution of mtDNA PCGs. Subsequently, we employ the branch model analyses to test our hypothesis. Analysis results show that there are seven rapid evolutionary genes in the large body size group and four rapid evolutionary genes in the medium body size group; however, in the small body size group, no genes exhibiting rapid evolution are identified (Table 1). Moreover, the small body size group exhibits significantly lower levels of ω values in three mtDNA PCGs. These results suggest a positive correlation between ω values of mtDNA PCGs and body size, indicating that body size may drive the molecular evolution of mtDNA PCGs in response to different energy requirements. Small-bodied species possess a higher surface area-to-volume ratio, which is particularly susceptible to losing heat at an alarming rate [58]. To combat this constant threat, they must maintain a heightened metabolic rate that allows them to generate enough energy. On the other hand, larger-bodied species are able to conserve energy more effectively and do not require the same level of metabolic efficiency as small-bodied ones [59]. As revealed by our research findings, larger-bodied species may experience relaxed functional constraints on their mtDNA PCGs following the degeneration of energy requirements. Due to most mutations having a negative impact [60], this is expected to be more pronounced in smaller-bodied species than in larger ones. Therefore, smaller-bodied species would have faced stronger evolutionary constraints in purging deleterious mutations and maintaining efficient energy metabolism.

Given the nearly universal negative correlation between body mass and effective population size (Ne), small-bodied species may display a more efficient purifying selection [61,62]. Thus, the lower ω values observed in small-bodied species could be attributed to a larger Ne [63]. The most direct approach to investigate the impact of Ne would be to compare the ω values between small-bodied and large-bodied species with similar Ne. Unfortunately, for most species, no direct estimates of Ne are presently available, but some work is still currently possible. Firstly, numerous studies have demonstrated that elevated/decreased ω values cannot be solely attributed to Ne; often, other forces are also at play [11,12,13]. Secondly, based on work by Chen et al. [64], we collect data on the Ne of 31 ruminant species over a period ranging from one million to ten thousand years (Supplementary Table S6). Analysis results reveal that there are no significant differences in the maximum, minimum, and mean values of Ne when compared with body mass (Supplementary Figure S1). Therefore, it can be inferred that there exists no discernible correlation between body mass and Ne among the species we studied. Third, the impact of Ne should be reflected in all loci throughout the genome. A comparative analysis was conducted on the ω values of 824 nuclear genes across different body mass in ruminants, revealing no positive correlation between body mass and ω value [64]. In summary, we believe that, apart from the influence of Ne, variations in constraints on energy metabolism account for differences in selective patterns observed in mtDNA PCGs of species with different body sizes.

The role of mitochondria in adaptive evolution has been widely proven [17,18,19,20,21,22,23,24]. Even in the context of strong purifying selection, positive selection may still occur for adaptation to environmental conditions. Based on the branch-site model analysis, we find one gene (*ND1*) that has undergone positive selection in the large body size group (Supplementary Table S5). We speculate that modifications to *ND1* observed in large-bodied species could be linked to certain adaptations unique to such body size. This suggests that different species may employ the same genetic toolkit to navigate environmental challenges [65].

Because species within the same body size group occupy different positions in the phylogenetic tree, our conclusions are not attributable to phylogenetic inertia. Nonetheless, we still perform PIC analysis, a powerful tool in evolutionary biology, to eliminate the confounding effects of phylogenetic inertia. Our regression analysis reveals a significant correlation between ω values of mtDNA PCGs and body mass (R^2^ = 0.162, *p*= 0.0016). Specifically, large-bodied species are associated with higher ω values, which is consistent with our main analysis.

## 5. Conclusions

In this study, we explore the relationship between molecular evolution of mitochondrial genes and body size in terrestrial Cetartiodactyla. The findings suggest that small-bodied species have experienced stronger purifying selection on their mitochondrial genes, while large-bodied ones may have undergone a relaxation of selective constraints due to lower metabolic efficiency. Despite the purifying selection being the predominant force in the evolution of mtDNA, we still found that several genes were under positive selection, suggesting the important role of mitochondrial genes in adaptive evolution. In short, our research provides valuable insights into the molecular evolutionary patterns of mitochondria genes.

## Figures and Tables

**Table 1:** The ω values of each mtDNA PCG among the three different body size groups through the branch model.

Gene	ω Values of Background	Large Body Size Group			Medium Body Size Group			Small Body Size Group		
		2ΔlnL	*p*-Value	ω Values	2ΔlnL	*p*-Value	ω Values	2ΔlnL	*p*-Value	ω Values
*ATP6*	0.03950	6.47440	0.01094	0.05896	1.57131	0.21001	0.03950	6.10818	0.01346	0.02852
*ATP8*	0.22740	0.62832	0.42797	0.22740	1.14865	0.28383	0.22740	0.30750	0.57922	0.22740
*COX1*	0.00369	0.02215	0.88169	0.00369	4.61687	0.03166	0.00542	2.58759	0.10770	0.00369
*COX2*	0.00773	7.03091	0.00801	0.01725	5.16766	0.02301	0.01175	9.30568	0.00228	0.00369
*COX3*	0.01707	6.55925	0.01043	0.03011	3.88503	0.04871	0.02271	0.00403	0.94934	0.01707
*CYTB*	0.02452	15.58788	0.00008	0.04042	13.57057	0.00022	0.03466	13.82839	0.00020	0.01616
*ND1*	0.01439	6.72214	0.00952	0.02233	0.75416	0.38516	0.01439	0.10258	0.74875	0.01439
*ND2*	0.03158	8.15511	0.00429	0.04675	0.02405	0.87676	0.03158	2.92590	0.08717	0.03158
*ND3*	0.02439	0.11558	0.73388	0.02439	1.14685	0.28421	0.02439	1.73338	0.18798	0.02439
*ND4*	0.02400	3.65796	0.05580	0.02400	0.38328	0.53585	0.02400	2.21415	0.13675	0.02400
*ND4L*	0.01654	0.15239	0.69627	0.01654	2.11266	0.14609	0.01654	1.03636	0.30867	0.01654
*ND5*	0.04655	13.96207	0.00019	0.06504	0.37257	0.54161	0.04655	0.32419	0.56910	0.04655
*ND6*	0.02791	1.78150	0.18196	0.02791	0.00447	0.94671	0.02791	0.41546	0.51921	0.02791

## Data Availability

All the mitochondria genome sequences used in this study were accessed through the GenBank database using the accession numbers in Supplementary Table S1.

## Supplementary Materials

The following supporting information can be downloaded at: https://www.mdpi.com/article/10.3390/ani14101434/s1, Table S1: Species information and GenBank accession number used in this study; Table S2: Information of species categorized based on body mass; Table S3: Data partitions and models identified by PartitionFinder; Table S4: The ω values of concatenated mtDNA PCGs and each mtDNA PCG of 59 species using the free-ratio model; Table S5: Test for positive selection on the three different body size groups based on each mtDNA PCG through the branch-site model; Table S6: Statistics on effective population size; Figure S1: Effective population size (Ne) exhibits no significant correlation with body mass.
