# Supplementary material for: Mitochondrial Genomic Evidence of Selective Constraints in Small-Bodied Terrestrial Cetartiodactyla
Source: Animals (Basel). 2024 May 10;14(10):1434. doi: 10.3390/ani14101434 (PMC11117313; doi:10.3390/ani14101434)
Supplement: Supplementary file 1 [file animals-14-01434-s001.zip › Supplementary Figure S1. Effective population size (Ne) exhibits no significant correlation with body mass.pdf]

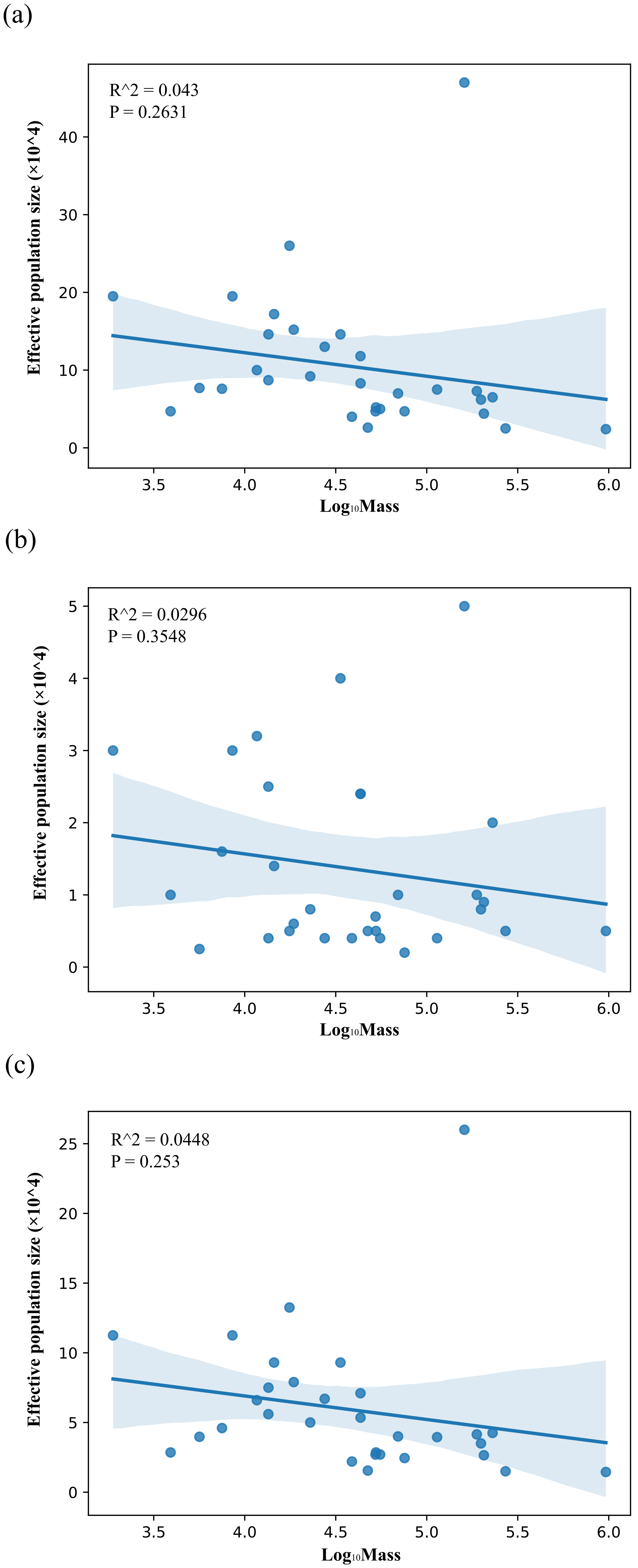

Figure S1. Effective population size (Ne) exhibits no significant correlation with body mass. Based on Chen, et al. inferred the demographic history for 31 ruminant species by applying the pairwise sequentially Markovian coalescence model. (a) The maximum N_e over a period ranging from 1 million to 10 thousand years in the past; (b) The minimum Ne over a period ranging from 1 million to 10 thousand years in the past; (c) The average Ne over a period ranging from 1 million to 10 thousand years in the past.
